# Depression of dynamic cerebral autoregulation during neural activation: The role of responders and non-responders

**DOI:** 10.1177/0271678X241229908

**Published:** 2024-02-01

**Authors:** Kannaphob Ladthavorlaphatt, Farhaana BS Surti, Lucy C Beishon, Thompson G Robinson, Ronney B Panerai

**Affiliations:** 1 Department of Cardiovascular Sciences, University of Leicester, Leicester, UK; 2 Medical Diagnostics Unit, Thammasat University Hospital, Thammasat University, Pathum Thani, Thailand; 3 Thammasat University Centre of Excellence in Computational Mechanics and Medical Engineering, Thammasat University, Pathum Thani, Thailand; 4 NIHR Leicester Biomedical Research Centre, British Heart Foundation Cardiovascular Research Centre, Glenfield Hospital, Leicester, UK

**Keywords:** Cerebral blood flow, cognitive stimulation, dynamic cerebral autoregulation, neurovascular coupling, reproducibility

## Abstract

Neurovascular coupling (NVC) interaction with dynamic cerebral autoregulation (dCA) remains unclear. We investigated the effect of task complexity and duration on the interaction with dCA. Sixteen healthy participants (31.6 ± 11.6 years) performed verbal fluency (naming-words (NW)) and serial subtraction (SS) paradigms, of varying complexity, at durations of 05, 30 and 60 s. The autoregulation index (ARI), was estimated from the bilateral middle cerebral artery blood velocity (MCAv) step response, calculated by transfer function analysis (TFA), for each paradigm during unstimulated (2 min) and neuroactivated (1 min) segments. Intraclass correlation (ICC) and coefficient of variation (CV) determined reproducibility for two visits and objective criteria were applied to classify responders (R) and non-responders (NoR) to task-induced MCAv increase. ICC values demonstrated fair reproducibility in all tasks. ARI decreased in right (RH) and left (LH) hemispheres, irrespective of paradigm complexity and duration (p < 0.0001). Bilateral ARI estimates were significantly decreased during NW for the R group only (p < 0.0001) but were reduced in both R (p < 0.0001) and NoR (p = 0.03) groups for SS tasks compared with baseline. The reproducible attenuation of dCA efficiency due to paradigm-induced NVC response, its interaction, and different behaviour in R and NoR, warrant further research in different physiological and clinical conditions.

## Introduction

Cerebral blood flow (CBF) is known to increase in response to neural stimulation, such as with cognitive paradigms, a mechanism usually referred to as *neurovascular coupling* (NVC).^1^ NVC processes take place via the neurovascular unit components to increase CBF through changes in the arteriolar and capillary diameter to match metabolic demand during neuroactivation.^2,3^ NVC has been extensively studied in both animals^4,5^ and humans,^1,6^ but little is known about its interaction with other CBF regulatory mechanisms, such as pressure autoregulation and vasoreactivity to changes in PaCO_2_. Cerebral autoregulation (CA) tends to maintain CBF approximately constant for a range of mean arterial blood pressure (MAP) values, that is narrower than originally proposed,^7^ as long as MAP does not change faster than approximately 0.2 Hz.^8,9^

The third mechanism that controls CBF, is CO_2_ VMR, whose interaction with CA is relatively well established. CO_2_ vasomotor reactivity (VMR) leads to vasodilation and increases CBF due to hypercapnia,^10^ which is known to worsen CA^11^ and to reduce NVC efficiency.^12,13^ On the other hand, little is known about the interaction of CA with NVC. Initial reports suggested that both visual^13^ and cognitive stimulation^14^ would lead to depression of dynamic CA (dCA). Further exploration of the NVC-CA interaction would be of considerable interest, not only to improve our understanding of these mechanisms, but also in clinical studies where assessment of either CA or NVC is relevant to the patient group of interest, and could be distorted by not taking their interaction into account. An interaction between NVC and CA could be anticipated for a number of reasons. Firstly, these mechanisms share common elements of the neurovascular unit, such as the role of astrocytes^15,16^ and common activation pathways, such as the involvement of the endothelium.^17^ Secondly, the end-result of either NVC or CA is the control of microvascular resistance, where the vasodilation induced by NVC stimulation might be either enhanced or depressed by corresponding CA responses, depending on the directional changes in BP.^18^ In a more recent study, Beishon et al. have confirmed that cognitive paradigms indeed reduced the Autoregulation Index (ARI), a well-established metric of dCA, but intriguingly, only in healthy subjects, and not in patients with Alzheimer’s disease (AzD) or mild cognitive impairment (MCI).^19^ The reasons for the differences in CA-NVC interaction in healthy versus cognitively impaired patients are not clear and the present study aimed to address some of the hypotheses stemming from those findings.^19^ First of all, the reproducibility of the tools used to express the interaction of CA with NVC have not been assessed and this is key for any interpretation of findings from studies in this area. This is of particular concern, mainly given the innovative use of short data segments to obtain ARI estimates, concomitantly with the presentation of cognitive tasks.^20^ Secondly, studies of NVC based on functional TCD (fTCD) have shown a dichotomy between responders (R) and non-responders (NoR) to a given cognitive test, and ignoring this factor could have influenced the results.^21^ Finally, another possible reason could be the nature of the cognitive tasks presented, involving repetitive subtraction and naming words starting with a fixed letter. The complexity and duration of these tests could also affect the interaction of NVC and dCA, which we took into consideration when designing this study. Also pertinent, is our choice of focusing on the middle cerebral artery (MCA) which supplies the frontal brain which is the region of the brain where we would expect increased metabolic demand due to the tasks selected. In summary, we tested three main hypotheses to address these potential factors affecting the CA-NVC interaction in healthy subjects, namely: i) the reduction in ARI due to neural stimulation is a reproducible phenomenon, despite the nature of the stimulation performed; ii) cognitive stimulation has different effects on the ARI of R vs NoR; and iii) the depression of dCA due to cognitive stimulation is dependent on the complexity or duration of the paradigms adopted.

## Materials and methods

### Subjects and measurements

All healthy participants were recruited from students and staff at University of Leicester, UK. Ethical approval was obtained from University of Leicester (ref: 19452-rp9-ls:cardiovascularsciences), and the study was performed according to the principles of the Declaration of Helsinki of 1975 (and as revised in 1983), and all subjects provided their fully informed and written consent before assessment. All subjects were free from any known cerebrovascular, cardiovascular, neurological, or respiratory conditions, including metabolic, inflammatory and severe somatic (e.g., cancer) or psychiatric (e.g., psychotic or bipolar) abnormalities. A detailed description of methods have been reported previously.^22^ The protocol consisted of two visits, using the same protocol, to allow assessment of reproducibility. The average time interval between visits 1 and 2 was approximately six months (170–232 days).

Subjects were asked to avoid strenuous exercise, caffeine-, nicotine- and/or alcohol-containing products, and large meals in the 4 h^23^ before arrival at the Cerebral Haemodynamics in Ageing and Stroke Medicine laboratory, a quiet environment, with controlled temperature (22–24°C) and lighting. Participants performed all measurements in a seated position with minimal movement in the same day, between 10 am to 2 pm for both visits. Cerebral blood velocity was measured bilaterally in the middle cerebral artery (MCAv) using 2-MHz TCD probes (DWL Doppler Box) at depths of 45–55 mm, with probes held in place with a headframe.^24^ Heart rate (HR) was measured by a three-lead electrocardiogram (ECG). End-tidal CO_2_ (EtCO_2_) and respiratory rate were monitored with a capnograph (Salter Labs, ref 4000, Capnocheck Plus) using nasal canulae. Beat-to-beat BP was continuously monitored with arterial volume clamping of the left middle finger artery (Finometer, Finapres Medical Systems; Amsterdam, the Netherlands). An automated brachial BP (UA767) device was used for measuring the systolic and diastolic BP before each recording. These values were used to calibrate the Finometer tracing. During editing, the entire Finometer signal was then calibrated by re-scaling all BP waveforms using as reference a stable early section of the recording. All physiological data were sampled at a rate of 500 samples/s and recorded onto a data acquisition system (PHYSIDAS, Medical Physics Department, University Hospitals of Leicester NHS Trust) for subsequent offline analysis.

The study design for presenting cognitive tasks of varying durations and complexity has been previously described.^25^ We have chosen naming words (NW) and serial subtraction (SS) for their ability to incorporate differences in cognitive load^25^ and to induce consistent MCAv responses^26,27^ as well as for their reproducibility.^28^ Moreover, both NW and SS are part of the current methods of cognitive assessment such as Addenbrooke’s Cognitive Examination (ACE-III), Mini Mental State Examination (MMSE) and Montreal Cognitive Assessment (MoCA).^29–31^ Baseline conditions were recorded for 5 min after resting (15 min). Then, subjects performed the two cognitive tasks of varying complexity and duration. Each measurement was recorded for a total of 3-min, including an initial 1-min period at rest where subjects were asked to relax and to maintain an ‘empty-mind’. During the next 1-min period, a single paradigm was presented and performed in random order, and the final 1-min period allowed recovery to the original baseline condition. Task complexity for the NW was increased with subjects asked to name as many words as possible, beginning with P-, R- and V- letters (NW) to represent increasing complexity,^32^ and with serial subtractions of 100-2, 100-7 and 1000-17 (SS), again reflecting increasing complexity. R-word and 100-7 were selected as the reference values for NW (NW_REF_) and SS (SS_REF_), respectively. The 5 s and 30 s epochs were used to test for the effect of changing the duration of stimulation. More detailed information of this aspect of the protocol, including a schematic representation of the timecourse of task presentation, can be found in.^22^

### Data analysis

All signals were visually inspected to identify artefacts or noise, narrow spikes (<100ms) were removed by linear interpolation. All signals were filtered with a low-pass, zero-phase eight order Butterworth filter with a cut-off frequency of 20 Hz. Smaller, narrow artefacts in bilateral MCAv signals were removed by a median filter. The R wave of the ECG signal was automatically detected for R-R interval calculation. The sequence of HR was visually inspected with manual correction of missing or misplaced marks. Beat-to-beat mean values of signals (MCAv, BP, HR) were calculated using the beginning and end of each cardiac cycle. Bilateral MCAv were expressed in percent (%) by normalising the 30 s of baseline before the paradigm activation. The end-tidal CO_2_ signal of each breath was detected in the capnographic tracing, linearly interpolated, and resampled with each cardiac cycle. All beat-to-beat parameter estimates were spline interpolated using a third-order polynomial and resampled at 5 Hz to generate a time series with a uniform time base.

### Autoregulation index

Transfer function analysis (TFA) can quantify the dynamic relationship between mean BP (MAP, input) and MCAv (output), assuming linearity and time-invariance. It also allows estimation of the ARI to express the effectiveness of dCA.^33,34^ However, instead of the recommended duration of 5 min, recordings of 3-minute duration were used^20^ during cognitive tasks. In this case, data segmentation for using Welch’s method^35^ employed segments with a duration of 51.2 s (256 samples). During baseline, conventional segments of 102.4 s (512 samples) were used. Each segment of data was multiplied by a cosine window, and the auto- and cross-spectrum were calculated with fast Fourier transform (FFT), using 50% superposition, as recommended in the CARNet white paper.^23,33^ TFA provides three parameters (gain, phase and coherence) that reflect the amplitude, time-shift and reproducibility of the relationship between MAP and MCAv, respectively. However, phase and gain estimates can only be considered reliable estimates of the relationship between MAP and MCAv at frequencies where coherence is above the upper 95% confidence limit.^19,33^ The impulse response was derived from the inverse FFT of the gain and phase, and was integrated to generate the MCAv step response to a sudden alteration of BP.^34^ The ARI value was estimated by the best fit of the MCAv step response with the 10 template curves proposed by Tiecks et al.^36,37^ Moreover, ARI estimates were only accepted if the average coherence function in the frequency interval 0.15–0.25 Hz was higher than the 95% confidence limit, and the normalised mean square error (NMSE) was below the critical limit of NMSE = 0.3 for fitting the MCAv step response to Tieck’s model.^38^

### Objective criteria for NVC response classification

Objective criteria were proposed by using the cross-correlation function peak (CCF) and variance ratio (VR) to classify subjects as R and NoR.^21^ In brief, the CCF can quantify the time-dependent correlation between individual signals and coherent averages of the MCAv response to stimulation.^39,40^ This can indicate that these signals will be highly correlated when CCF is approaching 1, but CCF approaching zero reflects absence of response. The VR represents the change in MCAv signal power from pre- to post-stimulation. The variant of the F-test was used as the ratio of two variances.^41^ Based on non-stimulated data, spontaneous fluctuations of MCAv at rest can provide the reference distributions of CCF and VR corresponding to the null hypothesis. Thus, applying the 90% confidence limits for CCF and VR (CCF_90_ and VR_90_) of the baseline ΔMCAv signals (unstimulated data), can be an objective threshold to classify subjects as R or NoR for each type of paradigm. Compared to the more commonly used 95% confidence limit, the 90% threshold provided greater sensitivity, to the detriment of specificity, and a better yield.^42^ For each task, a subject was classified as R if the response led to CCF/VR values greater than either CCF_90_ = 0.53 or VR_90_ = 2.59, as described previously.^21^ Subjects not meeting these criteria were labelled as NoR, for the given cognitive task.

### Statistical analysis

Normal distribution of data was assessed with the Shapiro-Wilk test. When no statistically significant differences were found, bilateral ARI values were averaged. The reproducibility within subject variation between two visits was expressed by the coefficient of variation (CV) calculated by dividing the SD by the mean value of the repeated measures. CV was interpreted as ‘very good’ (CV < 0.10), ‘good’ (0.10–0.20), ‘acceptable’ (0.20–0.30), or ‘not acceptable’ if CV > 0.30.^43,44^ Reproducibility was tested using the two-way mixed effect of intraclass correlation coefficient (ICC) with model (1,1) for absolute agreement between visits.^45^ Reproducibility was analysed according to the Cicchetti criteria: poor (ICC < 0.40), fair (ICC: 0.40–0.59), good (ICC: 0.60–0.74), and excellent (0.75–1.00).^46^ Two-way repeated measures ANOVA was used to evaluate the effect of visit and task on ARI estimates. When no significant differences were found between the two visits, ARI values for both visits were averaged. One-way repeated measures ANOVA or Friedman test was used to test for differences between haemodynamic parameters and ARI estimates during NW and SS paradigms in parametric or non-parametric data, respectively. Post-hoc analysis with Tukey’s or Dunn’s multiple comparisons was used when the ANOVA or Friedman test achieved statistical significance. Sample size calculation was based on our previous experience (n = 14) which demonstrated the presence of interaction.^14^ The effect sizes for pairwise comparisons^47^ and repeated ANOVA measures^48^ were calculated at 0.794 and 0.84, respectively. Data are presented as mean ± SD. Statistical analyses used GraphPad Prism Version 9.2.0 and SPSS version 25 for Windows. Significance was accepted at p < 0.05.

## Results

All subjects completed all cognitive paradigms of varying complexity and duration (NW and SS). Sixteen healthy participants (8 women; age 30.4 ± 4.1 years and 8 men; age 32.8 ± 16.4 years), with mainly right hand-dominance (87.5%), were enrolled in the study. Three participants were not able to attend the second visit and hence were removed from the assessment of reproducibility. At baseline, difference in physiological parameters between the two visits was only observed for HR (70.4 ± 8.1 vs 79.5 ± 9.6 bpm, p < 0.005). Mean right MCAv (62.0 ± 11 vs 57.4 ± 7.4 cm/s), left MCAv (61.0 ± 9.5 vs 62.4 ± 9.9 cm/s), BP (84.9 ± 11.3 vs 91.4 ± 12.1 mmHg) and EtCO_2_ (37.9 ± 3.1 vs 36.7 ± 1.5 mmHg) did not show differences between visits (all p > 0.08). Brain activation led to significant increases in mean bilateral MCAv, MAP, HR for both NW and SS. EtCO_2_ was reduced during NW, but not during SS (Table 1). Post hoc comparisons showed differences between baseline and each task activation (Table 1), but no differences due to the effects of NW and SS complexity and duration.

**Table 1.** Effects of NW and SS paradigms of different complexity on main physiological parameters.

Variables (n = 16)	Baseline	Activation (s)					p-value	Baseline	Activation (s)					p-value
		P-word (60 s)	R-word (05 s)	R-word (30 s)	R-word (60 s)	V-word (60 s)			100-2 (60 s)	100-7 (05 s)	100-7 (30 s)	100-7 (60 s)	1000-17 (60 s)	
RH.MCAv, %	−0.73 (0.9)	7.5 (5.8)^a^	5.2 (3.9)^a^	6.9 (4.9)^a^	6.1 (3.7)^a^	8.8 (5.3)^a^	<0.0001	−1.04 (1.0)	6.9 (4.0)^a^	3.7 (3.7)^a^	6.4 (3.7)^a^	4.1 (5.0)^a^	6.7 (5.7)^a^	<0.0001
LH.MCAv, %	−0.83 (0.7)	7.2 (6.0)^a^	5.8 (4.8)^a^	8.1 (4.8)^a^	6.7 (3.0)^a^	8.5 (3.4)^a^	<0.0001	−0.96 (0.9)	6.1 (2.7)^a^	3.8 (3.7)^a^	6.6 (4.5)^a^	4.2 (3.5)^a^	6.0 (1.8)^a^	<0.0001
MAP, mmHg	88.19 (12.6)	91.76 (13.0)^a^	97.11 (13.2)^a^	92.7 (12.0)^a^	92.56 (12.3)^a^	93.21 (13.5)^a^	<0.0001	88.56 (14.4)	91.85 (14.6)^a^	91.5 (13.4)	92.3 (14.0)^a^	92.56 (13.1)^a^	93.3 (15.4)^a^	0.0012
HR, beats/min	71.29 (8.5)	78.02 (7.6)^a^	76.1 (8.4)^a^	77.9 (8.9)^a^	78.05 (9.5)^a^	78.33 (10.0)^a^	<0.0001	71.18 (8.6)	76.85 (8.5)a	76.8 (7.3)^a^	77.8 (6.9)^a^	76.38 (7.4)^a^	76.49 (8.8)^a^	<0.0001
EtCO_2_, mmHg	37.29 (2.4)	36.74 (2.7)	36.6 (2.7)	36.0 (3.7)	35.68 (2.7)^a^	36.18 (3.0)	0.013	36.53 (3.0)	35.95 (4.1)	36.2 (3.4)	35.9 (3.1)	35.67 (3.0)	35.71 (3.2)	0.42

Values are mean (SD). MCAv: MCA cerebral blood velocity; MAP: mean arterial blood pressure; HR: heart rate; EtCO_2_: end-tidal CO_2_; RH: right hemisphere; LH: left hemisphere.

a Statistically significant difference from baseline for Tukey’s multiple comparisons at p < 0.05 after significance in one-way ANOVA.

### Reproducibility of ARI estimates between visits

**Figure 1.**
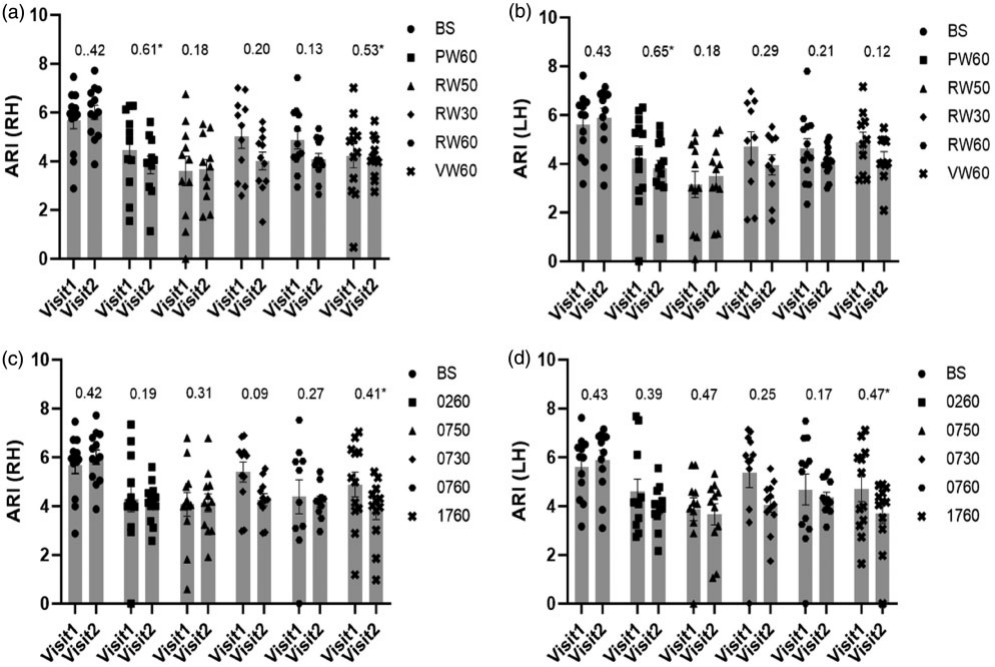
Population (range of n values = 10–13) mean ARI and SEM (error bar) with ICC values (above bars) for visits 1 and 2 during baseline (BS) and activation showing the lack of significant differences between the two visits. NW (a for RH and b for LH) and SS (c for RH and d for LH). ICC calculated using model (1,1). *Statistical significant ICC value (p < 0.05). 1(RH); right hemisphere, 2(LH); left hemisphere, BS; baseline, PW6; P-word 60 s, RW5; R-word 05 s, RW3; R-word 30 s, RW6; R-word 60 s, VW6; V-word 60 s, 026; 100-2 60 s, 075; 100-7 05 s, 073; 100-7 30 s, 076; 100-7 60 s, 176; 1000-17 60 s.

ICC values of ARI estimates, within visit agreement between left and right hemispheres, were excellent at baseline and all tasks with ICC > 0.76 (p < 0.001). Moreover, ICC values of ARI estimates were higher at baseline for NW tasks compared to the SS tasks, with most ICC values showing fair reproducibility (Figure 1). Significant ICC values were seen for P-word (RH = 0.61 and LH = 0.65) and 1000-17 (RH = 0.41 and LH = 0.47) for both hemispheres and V-word (RH = 0.53) at p < 0.044. On the other hand, both hemispheres (RH ICC = 0.42 and LH ICC = 0.43) and 100-7 (05 s) for LH (ICC = 0.47) provided p = 0.07, respectively. Although ICC values showed good and excellent reproducibility for V-word and P-word tasks, the R-word had ICC values indicative of poor reproducibility for all tasks of variable duration (<0.29 for bilateral hemispheres). For the within-subject variation of ARI estimates between two visits, mean CV values also provided acceptable and good reproducibility at baseline and task complexities with mean CV values higher for LH at R-word (05 s) = 0.32 and 100-7 (30 s) = 0.31, respectively (Table 2). On two-way repeated measures ANOVA (Table 2), ARI showed significant differences during neural activation in relation to the complexity of NW and SS (p < 0.0001), as well as duration of NW_REF_ and SS_REF_ (p < 0.0001) paradigms for each hemisphere, but no differences between visits (p = 0.17) or interaction of these effects (p > 0.06). Therefore, the averages of ARI values for both visits were presented as mean ARI at baseline and during paradigms for each hemisphere.

**Table 2.** Changes in mean ARI estimates due to task activation in both visits and hemispheres and means of their coefficients of variation CV with p-values from two-way ANOVA. The effect sizes for repeated ANOVA measures and pairwise comparisons were calculated at 0.84 and 0.794, respectively.

ARI					Effects (p-value)		
**NW(RH)**	**BS1**	**P-word**	**R-word**	**V-word**	Activation	Visit	Interaction
Visit1	5.8 (1.2)	4.7 (1.5)^a^	4.8 (1.4)^a^	4.2 (1.7)^a^	**<0.0001**	0.23	0.06
Visit2	6.0 (1.1)	3.9 (1.2)^a^	4.1 (0.8)^a^	4.1 (0.9)^a^			
mean CV	0.12	0.19	0.21	0.19			
**NW (LH)**	**BS2**	**P-word**	**R-word**	**V-word**	Activation	Visit	Interaction
Visit1	5.7 (1.2)	4.5 (1.8)^a^	4.6 (1.3)^a^	4.4 (1.7)^a^	**<0.0001**	0.29	0.34
Visit2	6.0 (1.3)	3.8 (1.2)^a^	4.1 (0.6)^a^	4.2 (1.0)^a^			
mean CV	0.13	0.20	0.18	0.23			
**SS (RH)**	**BS1**	**100-2**	**100-7**	**1000-17**	Activation	Visit	Interaction
Visit1	5.8 (1.2)	4.5 (1.8)^a^	4.5 (1.9)^a^	4.8 (1.8)^a^	**<0.0001**	0.33	0.15
Visit2	6.0 (1.1)	4.2 (0.8)^a^	4.2 (0.7)^a^	3.8 (1.3)^a^			
mean CV	0.12	0.17	0.22	0.25			
**SS (LH)**	**BS2**	**100-2**	**100-7**	**1000-17**	Activation	Visit	Interaction
Visit1	5.7 (1.1)	4.7 (1.6)	4.5 (2.0)^a^	4.5 (1.7)^a^	**<0.0001**	0.19	0.24
Visit2	6.0 (1.1)	3.9 (0.9)^a^	4.3 (0.7)^a^	3.7 (1.4)^a^			
mean CV	0.13	0.19	0.26	0.26			
**NW_REF_ (RH)**	**BS1**	**05s**	**30s**	**60s**	Activations	Visit	Interaction
Visit1	5.8 (1.2)	4.0 (2.0)^a^	5.1 (1.5)^b^	4.8 (1.4)	**<0.0001**	0.17	0.13
Visit2	6.0 (1.1)	3.7 (1.3)^a^	4.0 (1.2)^a^	4.1 (0.8)^a^			
mean CV	0.12	0.26	0.30	0.21			
**NW_REF_ (LH)**	**BS2**	**05s**	**30s**	**60s**	Activation	Visit	Interaction
Visit1	5.7 (1.1)	3.7 (1.9)^a^	4.8 (1.8)^b^	4.6 (1.3)^a^	**<0.0001**	0.35	0.33
Visit2	6.0 (1.1)	3.4 (1.4)^a^	4.0 (1.3)^a^	4.1 (0.6)^a^			
mean CV	0.13	0.32	0.22	0.18			
**SS_REF_ (RH)**	**BS1**	**05s**	**30s**	**60s**	Activation	Visit	Interaction
Visit1	5.8 (1.2)	4.2 (1.7)^a^	5.4 (1.4)^b^	4.5 (1.9)^a^	**<0.0001**	0.57	0.11
Visit2	6.0 (1.1)	4.1 (1.3)^a^	4.3 (0.8)^a^	4.2 (0.7)^a^			
mean CV	0.12	0.26	0.25	0.22			
**SS_REF_ (LH)**	**BS2**	**05s**	**30s**	**60s**	Activation	Visit	Interaction
Visit1	5.7 (1.1)	4.0 (1.6)^a^	5.3 (1.8)^b^	4.5 (2.0)^a^	**<0.0001**	0.26	0.09
Visit2	6.0 (1.1)	3.7 (1.4)^a^	4.1 (1.0)^a^	4.3 (0.7)^a^			
mean CV	0.13	0.28	0.31	0.26			

Values are mean (SD). CV: coefficient of variation; RH: right hemisphere; LH: left hemisphere; NW: naming words; SS: serial subtraction; BS: baseline.

a Significant difference with baseline for Tukey’s multiple comparisons at p < 0.05, ^b^significant difference due to duration of stimulation compared with 05 s within group at p < 0.05.

### Influence of responders and Non-Responders

**Figure 2.**
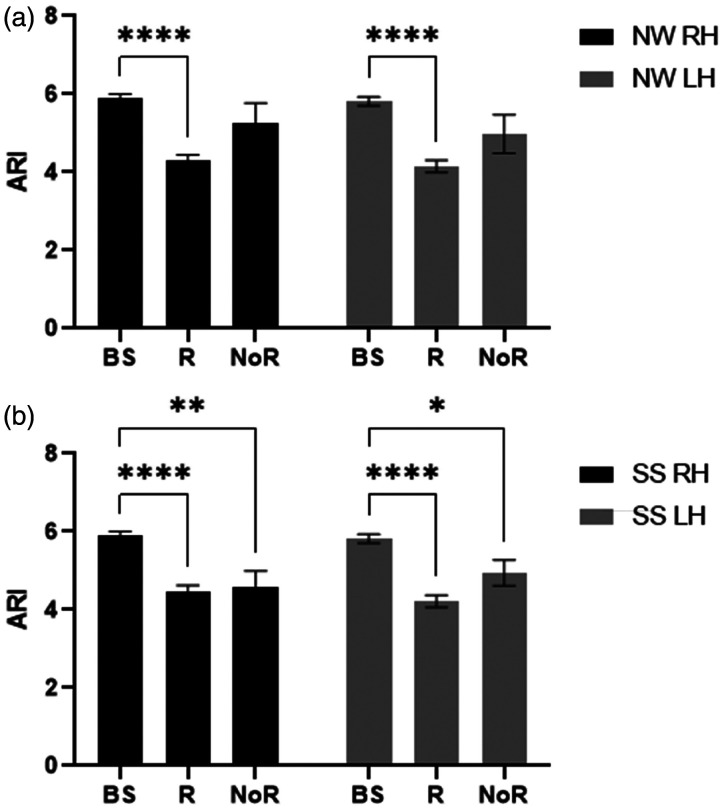
Population mean autoregulation index (ARI) and standard error of mean (SEM) (error bar) under objective criteria for responder (R) and non-responder (NoR) compared with baseline (BS) in right (black) and left (grey) hemispheres during naming words (a) and serial subtraction (b) tasks with the significance of post-hoc tests after 2-way ANOVA. *p < 0.05, **p < 0.01 and ****p < 0.0001.

CCF and VR criteria were applied to classify the number of R and NoR in each paradigm (Table 3). All individuals in the R group showed an increase in MCAv in at least one task. Moreover, bilateral ARI estimates in R were significantly decreased during NW paradigms (p < 0.0001) compared with baseline for RH and LH (Figure 2(a)). Likewise, bilateral ARI estimates were significantly reduced in R (p < 0.0001) and NoR (p = 0.03) groups during SS tasks compared with baseline (Figure 2(b)).

**Table 3.** Number (n = 16) and percentage of responders as classified by CCF_90_ and VR_90_ for MCAv_RH_ and MCAv_LH_ in averaged visits.

MCAv_RH_						MCAv_LH_				
**NW**	**P-60s**	**R-05s**	**R-30s**	**R-60s**	**V-60s**	**P-60s**	**R-05s**	**R-30s**	**R-60s**	**V-60s**
**R (%)**	15 (94%)	13 (81%)	13 (81%)	15 (94%)	16 (100%)	15 (94%)	13 (81%)	13 (81%)	16 (100%)	16 (100%)
**SS**	**2-60 s**	**7-05s**	**7-30s**	**7-60s**	**17-60s**	**2-60s**	**7-05s**	**7-30s**	**7-60s**	**17-60s**
**R (%)**	15 (94%)	12 (75%)	14 (88%)	16 (100%)	15 (15%)	14 (88%)	11 (69%)	13 (81%)	16 (100%)	11 (69%)

NW: naming words; SS: serial subtractions; MCAv: MCA cerebral blood velocity; R: responders; MCAv_RH_, MCAv_LH_: MCAv for the right and left hemispheres. P-60s; P-word 60 s, R-05s; R-word 5 s, R-30s; R-word 30 s, R-60s; R-word 60 s, V-60s; V-word 60 s, 2-60 s; 100-2 60 s, 7-05 s; 100-7 5 s, 7-30 s; 100-7 30 s, 7-60 s; 100-7 60 s, 17-60 s; 1000-17 60 s.

**Figure 3.**
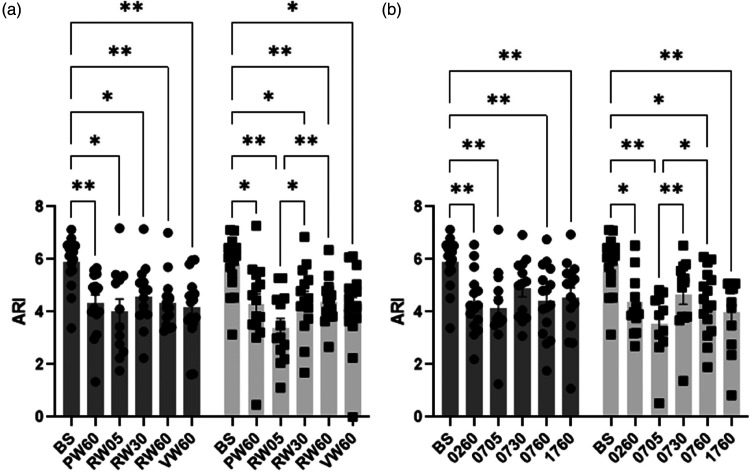
Population (range of n values = 11–16) mean autoregulation index (ARI) and standard error of mean (SEM) (error bar) during baseline (BS) and all naming words (a) and serial subtraction (b) tasks for R group in right (grey) and left (light grey) hemispheres. BS; baseline, PW60; P-word 60 s, RW05; R-word 05 s, RW30; R-word 30 s, RW60; R-word 60 s, VW60; V-word 60 s, 0260; 100-2 60 s, 0705; 100-7 05 s, 0730; 100-7 30 s, 0760; 100-7 60 s, 1760; 1000-17 60 s with post-hoc significance of 2-way ANOVA. *p < 0.05, and **p < 0.01.

Bilateral mean ARI estimates were determined for the R group only (Table 4), which were reduced during neural stimulation for all tasks of NW, SS, NW_REF_ and SS_REF_ (p < 0.0001). In post-hoc analyses, all complexity levels and durations of NW (Figure 3(a)) and SS (Figure 3(b)) tasks showed a significant decrease of bilateral ARI values from baseline (p < 0.03), with the exception of SS_REF_ (30s) for both hemispheres (p = 0.09). However, no significant differences in bilateral ARI values were seen between each task irrespective of complexity, with the exception of SS_REF_ at 05 s showing a significant decrease in ARI value for LH when compared to 30 s and 60 s (p = 0.03).

**Table 4.** Effect of cognitive tasks of different complexity and duration for responders only on the mean ARI of two visits.

ARI							Effects (p-value)		
**NW**	**BS**	**P-60s**	**R-05s**	**R-30s**	**R-60s**	**V-60s**	Activation	Hemisphere	Interaction
RH	5.9 (1.0)	4.4 (1.2)^a^	4.0 (1.7)^a^	4.6 (1.2)^a^	4.3 (1.0)^a^	4.2 (1.3)^a^	**<0.0001**	0.16	0.66
LH	5.8 (1.1)	4.3 (1.6)^a^	3.4 (1.3)^a^	4.4 (1.3)^a^^b^	4.4 (0.8)^a^^b^	4.2 (1.5)^a^			
**SS**	**BS**	**2-60s**	**7-05s**	**7-30s**	**7-60s**	**17-60s**	Activation	Hemisphere	Interaction
RH	5.9 (1.0)	4.4 (1.2)^a^	4.2 (1.5)^a^	4.9 (1.1)	4.4 (1.3)^a^	4.5 (1.5)^a^	**0.0001**	0.07	0.26
LH	5.8 (1.1)	4.4 (1.2)^a^	3.5 (1.3)^a^	4.7 (1.4)^b^	4.4 (1.2)^a^^b^	4.0 (1.4)^a^			

Values are mean (SD) with two-way ANOVA. RH: right hemisphere; LH: left hemisphere; NW: naming words; SS: serial subtraction; BS: baseline.

a (p < 0.05) compared to baseline for Tukey’s multiple comparisons.

b (p < 0.05) due to duration of stimulation compared with 05 s within group.

### Inter-hemispherical differences

Overall, no significant interhemispheric differences were seen during NW (p = 0.16) and SS (p = 0.07) tasks (Table 4). There were no interhemispheric differences influenced by either complexity or duration of cognitive paradigms on ARI estimates.

## Discussion

The present study has contributed three main findings. First, we demonstrated consistency in reduced dCA responses across two visits to both NW and SS paradigms. Second, the influence of R and NoR on ARI estimates was seen with NW tasks, but not for SS tasks. Third, an attenuation of the dCA response during NVC activation, irrespective of cognitive load, represented by different task complexity or durations of stimulation. These findings have confirmed our initial hypotheses and are in line with previous studies.^14,49^

### Reproducibility

Poor reproducibility of dCA parameters has been reported in previous studies due to the low power spectral density of mean BP^50–52^ or the effects of physiological variability on the BP-MCAv relationship.^53^ This study demonstrated no significant effect of visits (Table 2), but the ICC was not always as high as expected, particularly during neural activation (Figure 1). At rest, a well-maintained cerebral perfusion and efficient dCA would be expected in healthy subjects, but this does not necessarily imply elevated values of ICC. The determinants of physiological variability and low power spectral density of mean BP could be behind the low ICC value of ARI estimates observed during task-activated NVC responses. Nevertheless, further investigation is warranted to determine if ICC values could be improved by enforcing more rigorous external conditions, such as minimizing noise and distractions^33^ or removing recordings with low power spectral density of mean BP.^52^ Despite good reproducibility for cerebral blood velocity (CBv) response to chosen NW and SS tasks,^28^ the poor reproducibility of R-NoR classification estimates would be confounded by physiological variables on each day of recording such as BP, HR and respiratory sinus arrythmia (RSA)-induced EtCO_2_ fluctuations. Hence, the reproducibility between recording days may be further considered to address the effect of physiological fluctuations.

### Classification criteria on dCA estimates

Objective criteria were used to distinguish between R and NoR on TCD-measured MCAv responses to express the integrity of NVC mechanisms.^21^ Many previous NVC studies were limited to peak percentage change of MCAv signals.^26,28,54–56^ Subjective classification of MCAv changes might fail to exclude non-responders and lead to distortions in the population average.^56^ This limitation was overcome by utilising the CCF and VR estimates to provide objective criteria to improve classification and lead to more robust inter-study comparisons. However, the distribution of NoR showed some inconsistency (Table 3), which might be a random effect between tasks and participants. When considering the influence of R and NoR with NW and SS tasks on ARI estimates, we would expect NoR to be less affected by cognitive activation when compared to R subjects in each task. This expectation was confirmed for NW paradigms (Figure 2(a)) but one interesting finding, was that during SS paradigms, the ARI was also reduced for NoR (Figure 2(b)). This differential response to the two types of paradigms is relevant to a better understanding of the dCA-NVC interaction and deserves further investigation. Given that the number of NoR classifications were approximately the same for both NW and SS (Table 3), it is more likely that the different behaviour of the NoR group for SS paradigms could be due to differences in dCA for these subjects. One possibility would be the larger increase in BP observed with NW stimulation, compared to SS paradigms (Table 1). With a reduced demand for vasoconstriction during SS stimulation, due to the lesser increase in BP, the NoR subset could be more sensitive to the demand for vasodilation, thus leading to a reduction in ARI, similar to the R group.

### dCA during neural stimulation

Brain activation paradigms (neural or visual stimulation) have demonstrated significant changes in TFA parameters, suggesting reduced dCA efficiency.^13,14,49^ In agreement with our present findings, attenuated ARI values were observed during NW and SS paradigms of varying complexity and duration. It has been suggested that metabolic demand-induced cerebral vasodilatation resulting from cognitive stimulation affects the efficiency of dCA through cerebral vasomotion.^49^

The pattern of MCAv response to BP fluctuations and neural activations depends on a balance of vasoconstriction (myogenic) and vasodilatation (metabolic) changes, similar to hypercapnia. The corresponding changes in the diameter of small cerebral arteries and arterioles, will alter CBF, but as long as the MCA diameter remains constant, MCAv will still provide a reliable indication of these changes.^11,57,58^ In our results, neural activations provoked significant increases in bilateral MCAv and BP, while reduction in EtCO_2_ was associated with reduced averaged responses to R-words at 60 s stimulation (Table 1). Also, cognitive stimulation can induce hyperventilation, thus leading to hypocapnia,^1,59,60^ rather than hypercapnia, which may enhance dCA efficiency during neural activation. However, our findings indicate that neuronal activation attenuated dCA capacity, as reported in several clinical studies such as MCA stenosis,^61^ ischaemic stroke^62^ and subarachnoid haemorrhage.^63^ This may result from temporary impairment in vascular smooth muscle cell contraction in response to BP changes. These findings indicate the preponderance for cerebral metabolic demand as the primary mechanism responsible for the observed attenuation of cerebral autoregulatory function. The balance between myogenic and metabolic mechanisms during neural activation during NO-induced vasodilation has been demonstrated in a recent study with a single arteriole model.^64^ The cerebrovasculature is known to be richly innervated by both sympathetic and parasympathetic branches of the autonomic nervous system ^65^ and its involvement could have contributed to the attenuation in dynamic CA that we have observed during task activation. The dominant view in the literature is that sympathetic activation leads to vasoconstriction and protects the brain during rapid increases is BP,^66^ whilst vagal activity can also lead to alterations in CBF.^67,68^ In our study, there were increases in MAP and HR, in response to task activation (Table 1), that could be explained by the sympathetic response to a stress reaction. Whether similar changes in sympathetic activity were also affecting the brain vasculature, is still open to speculation,^69^ but if this happened, it would be one more source of interference with the myogenic response that should be the dominant mechanism during dynamic CA. In summary, without other interferences, dynamic CA would be responding only to rapid changes in MAP, leading to vasoconstriction or vasodilation of the microcirculation. However, in the presence of metabolic or neurogenic inputs also competing to influence vascular smooth muscle activity, it is likely that the myogenic pathway will be undermined thus leading to reduced dynamic CA effectiveness, as expressed by the ARI. A multivariate model including the different inputs influencing the MCAv response is warranted to further investigate the relationship between dCA efficiency and neural activations, to improve our understanding of the different relative contributions of intervening factors.

The largest decreases in ARI caused by neural stimulation were induced with the shortest (05 s) duration of both NW_REF_ and SS_REF_ from baseline (Table 2, p < 0.004, Tukey’s post hoc test). It has been demonstrated that ARI can be depressed at the beginning of hyperventilation due to an alert reaction.^70–72^ Therefore, dCA capacity might be reduced by the temporal effect of metabolic demand with a short duration of stimulation. Furthermore, a high velocity subcomponent due to critical closing pressure (V_CrCP_) (possibly representing metabolic activation)^54,73^ response was seen in the shortest (05 s) duration paradigm in our previous study,^22^ concomitantly with the lowest ARI estimates for these tasks. These findings suggest thought processes with a short duration during the stream of consciousness might be impacting the results. Although attention and fluency tasks induced a reduction in ARI values in the present study, these tasks have not shown significant reductions in ARI estimates in healthy older adults as seen in our more recent study.^19^ It is more likely that the changes of interaction between dCA and NVC in response to task activation might be as a result of age-related changes in cerebral vasomotion.^42,74^ However, the reduction of ARI values due to mental activation is highly relevant to improve our understanding of dCA nonstationarity^75^ with its inherent physiological variability, which might be the predominant cause of poor reproducibility^53,76^ seen in task-activated studies. Thus, the BP-MCAv relationship may rapidly shift from the linear nature of dCA^77^ at rest to nonlinear behaviour during neural stimulation, due to the interaction between myogenic and metabolic activities. Based on these findings, short duration paradigms could be advantageous for task-activated studies in healthy or diseased conditions to reduce its effects on dCA. One could also anticipate that episodes of mental activation might precipitate the occurrence of syncope through reduced dCA efficiency over a short period of time.^78^

### Methodological considerations

This is the first study to assess dCA-NVC interaction showing decreased ARI during cognitive tasks using short segments of data.^20^ Our results agree with previous studies, based on longer recordings of at least 5 min to obtain dCA metrics for TFA,^13,14,49^ but provides a much broader perspective on the interaction between dCA and NVC mechanisms.

The reproducibility of ARI estimates derived from shorter segments of data could be affected by the necessary reduction in the number of superposing data windows,^35^ compared to the 5-min standard.^33^ However, signal segmentation with different window lengths were demonstrated to provide consistent estimates of phase and gain values at low frequency (LF),^79^ as well as TFA-derived ARI estimates,^38^ and in the comparison of the ARI between healthy subjects and patients with ischaemic stroke.^80^ Therefore, we limited the data segments to 3 min using 51.2 s duration of each segment (256 samples) to improve the sensitivity of ARI estimates, considering that the longest cognitive stimulations lasted only 60 s. Of note, reducing the duration of the data segments used to estimate the ARI requires a corresponding change in the 95% upper confidence limits for the mean coherence in the 0.15–0.25 Hz frequency range, to maintain the reliability of ARI estimates derived from the MCAv step response via the inverse FFT of gain and phase.^38,80^ In the worst case of MCAv overestimating changes of CBF, these would affect gain (amplitude) but not the coherence and phase of TFA due to these parameters not being sensitive to amplitude changes.^14^ Hence, gain would only affect the amplitude of the response and corresponding values of ARI would not be affected.

### Clinical implications

The interaction between dCA and NVC that we and others have described in healthy subjects^13,14,49^ have also been reported in a few clinical studies.^19,81,82^ In some studies of ischaemic stroke, both dCA and NVC were shown to be depressed when assessed separately.^83,84^ The absence of dCA-NVC interaction in patients with cerebrovascular conditions could be the result of impairment in one of these mechanisms, without alterations in the other. Auditory comprehension tasks were used to activate auditory cortex induced haemodynamic responses in acute (within 4 hours) and subacute (5–12 days) ischaemic stroke, and dCA was assessed with mean flow index (Mx), resulting in increased Mx values (deterioration of dCA capability) for bilateral hemispheres between acute and subacute phases, with a significant difference only in the affected hemisphere.^81^ This result could imply that the dCA response was diminished due to neural activation by temporally inhibiting myogenic tone to activate mechanically sensitive ion channels and proteins in the vessel wall on the affected side by longitudinal changes. More recently, cognitive tasks have been employed to assess the dCA-NVC interactions in patients with cognitive impairment, showing a significant attenuation of ARI estimates in older healthy adults with language and memory tasks, but not in MCI and AzD patients.^19^ Thus, deterioration of metabolic relative to myogenic mechanisms occurs in cognitively impaired populations. Hence, the interaction between dCA and NVC activation with cognitive tasks needs further investigation in clinical populations with variable cognitive tasks and conditions such as dementia or cerebrovascular dysfunction. These might improve our understanding in their interactions with regulatory impacts on the cerebrovascular tone.

### Limitations

Several limitations of this study need to be considered. Firstly, reliable CBF estimates with TCD-measured MCAv are based on the assumption that the cross-sectional area of the MCA remains relatively constant despite small changes in BP or PaCO_2_.^85,86^ Nevertheless, more recent studies have only found changes in the cross-sectional area with very high values of EtCO_2_.^87^ This could be a concern in the significant decrease in EtCO_2_ observed during the R-word paradigm (Table 1), and in mental activation increased sympathetic activation (high neurogenic response). However, significant changes in the cross-sectional area do not tend to arise during cognitive tasks, as a confounding factor^85,88^ given the ranges of EtCO_2_ observed in these studies. Secondly, as expected with a single presentation for each task, due to lower signal to noise, poor ICC and unacceptable CV were obtained as typical errors of responses when objective measures to identify ‘R’ and ‘NoR’ to NVC cognitive paradigms. This is an evolving challenge that still needs much more research and future studies. Thirdly, we have used only NW and SS tasks,^22,25^ corresponding to fluency and attention cognitive domains, to study the interaction between dCA response during neuroactivation with tasks of variable complexity and duration. These tasks have been used in several previous studies^26,27^ and the fact they are part of a widely used clinical tool^30^ support their choice to further standardise NVC methods across studies and research centres. Nonetheless, different types of neural stimulation, of variable complexity and duration (motor, auditory or visual) should also be considered to assess the dCA response associated with the varying levels of cerebral metabolic demand. Fourthly, this study was limited to the insonation of the MCA only, considering that it provides 80% of cortical blood supply.^89^ However, the MCAv responses might not provide similar sensitivity with greater spatial resolution (PET or fMRI) to cognitive stimulation.^1^ Therefore, other arteries, such as the anterior or posterior cerebral arteries, might demonstrate better insights on the dCA response to paradigms of varying complexity and duration. Moreover, the activation of the nucleus basalis modulates globally cortical blood flow through an intrinsic cholinergic mechanism, and serotonergic and α2 adrenergic receptor-mediated dilation may involve locus coeruleus which are relevant to neural circulation, neural plasticity, and cognition.^90^ This might affect increases in global MCAv during cognitive task activation. Fifthly, since the number of NoR in each task stimulation was small, we could not perform statistical analyses to compare between R and NoR for each task. Sixthly, we only analysed the reproducibility of measurements between the two visits, to decide on the feasibility of using averaged values from the two visits, based on the results from the ANOVA, rather than being informed by the ICC. Using averaged values from the two visits was assumed to provide more robust results to test the main hypothesis of the study, but further separate analyses of each visit are warranted. Finally, we did not control for the phase of the menstrual cycle in our female subjects. Estrogen levels might have affected the cerebral myogenic response through both endothelium-derived cyclooxygenase and nitric oxide synthase-dependent mechanisms,^91,92^ although these effects are still controversial.^93^ The study of the effect of age and sex on CBF regulation in humans should be a priority in future studies. Moreover, future studies will also have to address the complex interaction between motor activation and its related cognitive load during speech as part of the feedback provided by participants as they respond to the NW and SS paradigms. The design of specific protocols for this purpose are likely to be very elaborate and beyond the scope of the present investigation. Of note, this additional component of the overall metabolic demand induced by task activation is likely to be constant for all the six different paradigms that we employed.

## Conclusion

We conclude that dCA is temporally depressed during cognitive activation paradigms, based on ARI estimates obtained from TFA with objective criteria for classifying R and NoR. For NW tasks, the dCA-NVC interaction was present in responders, differently from NoR, but this separation was not observed with SS tasks. The reduction in ARI values was also seen in the shortest duration of stimulations, compared with longer durations or resting, irrespective of complexity or duration. Further studies are needed to extend our findings to different cerebrovascular conditions, other types of paradigms, and to assess the effects of sex and ageing on this phenomenon.
